# Treatment with recombinant human bone morphogenetic protein 7 leads to a transient induction of neutralizing autoantibodies in a subset of patients

**DOI:** 10.1016/j.bbacli.2016.08.001

**Published:** 2016-08-03

**Authors:** Andrea Schuette, Arash Moghaddam, Petra Seemann, Georg N. Duda, Gerhard Schmidmaier, Lutz Schomburg

**Affiliations:** a Institute for Experimental Endocrinology, Charité - Universitätsmedizin Berlin, D-13353 Berlin, Germany; b Department of Orthopedics and Trauma Surgery, Heidelberg University Hospital, D-69118 Heidelberg, Germany; c Berlin-Brandenburg Center for Regenerative Therapies (BCRT), Charité - Universitätsmedizin Berlin, D-13353 Berlin, Germany; d Julius Wolff Institute, Charité - Universitätsmedizin Berlin, D-13353 Berlin, Germany

**Keywords:** Autoimmunity, Consolidation, Biologicals, Fracture, Growth factor, Bone morphogenetic protein

## Abstract

**Background:**

Recombinant human bone morphogenetic protein 7 (rhBMP7) is applied for treatment of bone fractures, especially tibial non-unions. Its application may induce autoantibodies (aAB) affecting the targeted and endogenous signaling pathways and in turn negatively impact treatment efficacy.

**Methods:**

Novel and sensitive assays for the quantification of BMP7-aAB and BMP2-aAB were established and used to analyze serum samples from healthy controls (n = 100 men, n = 100 women) and patients with long bone fracture (n = 265) treated or not with rhBMP7. Sera from three to nine time points per patient were available and enabled the evaluation of aAB over a time course of up to one year. Functional activity of the BMP-aAB was tested with a BMP-responsive cell-based reporter assay. Consolidation of the fracture was evaluated as clinical outcome potentially affected by BMP7-aAB.

**Results:**

Prevalence of BMP7-aAB and BMP2-aAB was 1–2.5% in non-treated patients or healthy controls. The rhBMP7 treatment induced a transient increase in BMP7-aAB in a subset of patients, returning to non-detectable levels within six months. IgG from BMP7-aAB positive sera inhibited dose dependently the BMP7-reporter gene activity, whereas control sera were without effect. Successful consolidation of the fracture was observed in the majority of both aAB-positive and aAB-negative patients.

**General significance:**

We conclude that BMP7-aAB can be detected as natural aAB in healthy subjects, and are transiently induced by rhBMP7 therapy in a subset of patients. The aAB are capable of antagonizing BMP7 signaling in vitro, but do not preclude treatment success in patients.

## Highlights

• There are patients with natural autoantibodies recognizing BMP7.
• In some patients, rhBMP7-therapy induces BMP7 autoantibodies.
• BMP7 autoantibodies elicit neutralizing effects on BMP signaling.
• Therapy-induced BMP7 autoantibodies disappear over time.
• BMP7 autoantibodies seem not to affect therapy success.

## Graphical abstract

Statement of significance: The treatment of bone fractures with recombinant human BMP7 (rhBMP7) induces a transient peak of autoantibodies to BMP7. These antibodies are active as antagonists in vitro, but do not preclude treatment success. These findings are of relevance to rhBMP7-based treatments and the interpretation of health risks by drug induced autoantibodies.

**Figure:**
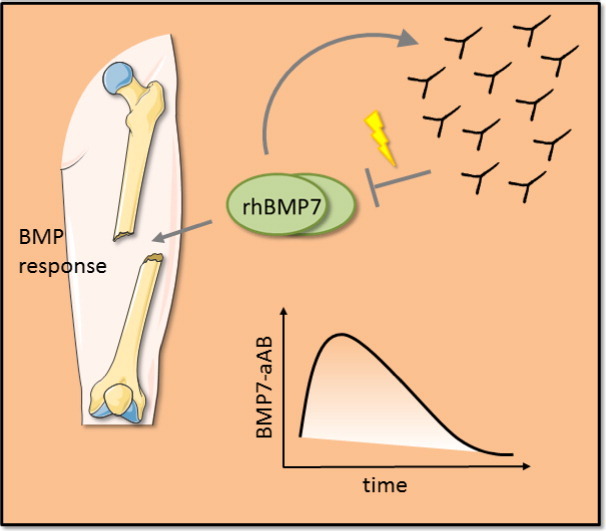

## 1 Introduction

Bone is a tissue with a remarkable regenerative potential controlled in part by bone itself and by the interplay with the immune and vascular systems [1]. After fracture, bone often regenerates completely to its original composition without the formation of a scar. It can therefore be considered as a truly regenerative tissue. Usually, a fracture gap is closed within 3–6 months after trauma. However, some fractures (approx. 10%) show healing difficulties leading to delayed healing, or non-unions also known as pseudarthrosis. There are a number of parameters affecting the healing process including the severity of the initial insult as well as age and health of the patient [2], [3], [4].

A compromised healing situation requires an intervention to reactivate and enhance natural bone formation. The major aspects that need to be considered are osteogenic cells, osteoconductive scaffolds, osteoinductive stimulants (hormones and local growth factors) and the mechanical environment, summarized as the diamond concept [5] which was extended by the aspect of vascularity [6]. Interventions according to the diamond concept involve an assessment of all of these aspects for a given patient and the attempt to optimize the therapeutic measures resulting in an individualized therapy plan. Treatment of non-unions following this concept proved to be a reasonable and successful strategy [7], [8], [9]. In early stage the therapeutic treatments in delayed union may include biophysical stimulation, e.g. full weight bearing, low-intensity pulsed ultrasound, shockwave or electromagnetic field stimulation. Biological enhancement of bone regeneration is the base in treatment of non-unions. Autologous cancellous bone graft is considered the gold standard in the surgical treatment of non-unions, but the limited availability is problematic [15]. Amongst the locally applied biological enhancers are calcium phosphate or collagen sponges as osteoconductive material, growth factors like erythropoietin, fibroblast growth factors or bone morphogenetic proteins (BMPs) as osteoinductive agents and synthetic polymers or autologous bone as osteogenic material [10].

BMPs belong to the transforming growth factor beta (TGFβ) superfamily and are pleiotropic paracrine growth factors that are involved in the regulation of diverse biological processes such as proliferation, survival, apoptosis, differentiation and migration of cells [11]. The different members of the TFGβ-superfamily perform specific tasks during development and homeostasis in various tissues [12]. The groundwork of BMP research in bone was laid in the late 1960s by Marshal Urist when he showed that implanted demineralized bone induced ectopic bone formation in skeletal muscle [13]. Later, the nature of these bone forming factors were identified and termed BMP [14]. The corresponding DNA was cloned and recombinant protein was expressed shortly after [15].

These achievements paved the way for applying recombinant human BMP (rhBMP) to improve bone regeneration and fracture healing. To this end, especially rhBMP2 and rhBMP7 have been tested and further developed to therapeutic biologicals as osteoinductive growth factors and work most efficient in combination with autologous bone material [16], [17], [18], [19]. BMP2 and BMP7 induce osteoblast and chondrocyte differentiation thereby increasing intramembranous and endochondral ossification. Both BMPs have been approved for use in humans by the FDA in 2001 and 2004, respectively, and have shown remarkable therapeutic effects in the last decade. However, concerns regarding their safety and side-effects were raised, especially with respect to ectopic bone formation, osteolysis, induction of autoimmunity, cancer, or problems related to cost effectiveness, collectively limiting their use in recent years [20].

The potential induction of autoantibodies (aAB) against rhBMP (BMP-aAB) may be of clinical relevance for three major reasons. First, BMP-aAB might interfere with the biological activity of the therapeutic rhBMP by neutralizing it. Second, rhBMP-aAB complexes may cause unwanted immune reactions. And third, treatment-induced BMP-aAB might cross-react with endogenous BMP thereby interfering with the regular signaling pathways.

In order to test for natural occurring and treatment-induced BMP-aAB, we developed two novel luminometric assays. We determined the prevalence of BMP7-aAB and BMP2-aAB in healthy subjects and in patients with severe fractures treated or not with rhBMP7, and at time points before and after surgery. Our data indicate that rhBMP7 treatment transiently induces BMP7-aAB in a subset of patients, and that these aAB are antagonists for BMP7 signaling in vitro. However, fracture consolidation was successfully achieved in both BMP7-aAB-positive and BMP7-aAB-negative patients, indicating that these aAB do not preclude treatment success.

## 2 Material and methods

### 2.1 Patients

Serum samples from a cohort of 200 anonymized healthy donors (100 males and 100 females, age range; 21 to 40 years) were obtained from a commercial supplier (Invent GmbH, Biotechnology Center Hennigsdorf, Germany). Serum samples from fracture patients were collected at the Department of Orthopedics and Trauma Surgery, Heidelberg University Hospital. Two time points were analyzed from 265 patients with long bone fracture (189 females, 76 males), yielding a total collection of 530 samples. The first time point was around surgical intervention (either pre-surgery or two days after surgery), and the second time point was approximately four weeks after surgery. The patients were categorized into different groups according to whether they have been treated with rhBMP7 or not (Table 1).

The study was conducted in accordance with the declaration of Helsinki. All individuals provided written consent to the study protocol. The study was approved by the ethics committee of the Ruprecht- Karls-University of Heidelberg (S-636/2011).

### 2.2 Quantification of BMP7-aAB and BMP2-aAB

The rhBMP7 used in the osteoinductive therapeutic intervention was used as bait to establish a novel assay for detection and quantification of naturally occurring and therapy-induced aAB against BMP7. For reasons of testing the specificity, a second analogous assay for aAB against BMP2 was established. To this end, 0.1 mg of collagen-free rhBMP7 (Olympus Biotech) or rhBMP2 (Metronic) were labelled with acridiniumester-*N*-hydroxy-succinimid (MACN, InVent Diagnostica GmbH) in an amine-free buffer. The labelling reactions were stopped by adding 1 M Tris, pH 7.5. The MACN-labelled rhBMP7 or rhBMP2 was diluted in buffer (PBS, 1% BSA, 0.1% NaN_3_) and separated from unbound MACN using 10 kDa MWCO centrifugal filter units (Centricon Ultracel-10K, Millipore, Eschborn, Germany).

After optimizing the conditions of BMP7- or BMP2-aAB detection and quantification, the following protocol was established and used throughout this study. Serum samples (10 μl per reaction) were incubated with diluted MACN-labelled rhBMP2 or rhBMP7 (100 μl per reaction) and incubated at 4 °C overnight. The next day, IgG were bound by incubation for 1 h shaking at 300 rpm at room temperature with a solution of a 10% protein A slurry in PBS (PorosA®, 50 μl, Applied Biosystems). The samples were washed three times with 1 ml washing buffer (50 mM KH_2_PO_4_/K_2_HPO_4_ pH 7.5, 100 mM NaCl, 0.1% TritonX-100), the pellet was precipitated by centrifugation (5 min, 3500 rpm, 20 °C) and the supernatant was aspirated. The chemiluminescence of the bound MACN-labelled rhBMP2 or rhBMP7 was measured in a chain luminometer (Autolumat Plus LB 953, Berthold Technologies, Bad Wildbad, Germany). For the characterization of the assay and as positive controls, anti-BMP7 and anti-BMP2 antibodies were used. Mice were immunized with rhBMP7 and monoclonal anti-BMP7 antibodies were generated by a commercial partner (UNICUS Karlsburg OHG, Greifswald, Germany). A commercial anti-BMP2 antibody (CYT-26591) was purchased (Dianova, Hamburg, Germany). Both antibodies were applied in a concentration of 100 μg/ml in the control experiments.

### 2.3 Isolation of IgG

Total IgG of BMP7-aAB positive and negative sera were isolated by precipitation with protein A. Serum samples (300 μl) were incubated with a slurry of 50% PorosA® in PBS (600 μl) and incubated overnight at 4 °C under constant agitation. The supernatants were discarded and the pellets were washed six times with PBS. Precipitated IgG were eluted with 25 mM citric acid, pH 2.2. Seven fractions (500 μl each) were collected and neutralized by addition of 1 M HEPES, pH 8.0. The volume of the eluate was reduced by spinning in centrifugal filtration units (Centricon YM-50, Amicon, Millipore), adjusted to a volume of 300 μl and stored at 4 °C until use.

### 2.4 BMP-reporter assays

To test for potential effects of BMP-aAB on BMP signaling in vitro, we used a BMP-responsive-element (BRE)-containing reporter plasmid, kindly provided by Prof. Dr. Peter ten Dijke [21]. NIH3T3 cells (ATCC® CRL-1658™) were cultured in standard medium (DMEM/F12, 10% FBS). Cells were seeded on 96 well assay plates (white, clear bottom, Corning Incorporated, NY, USA) at 10,000 cells per well. The next day, cells were transfected with the BRE reporter and a control plasmid of secreted alkaline phosphatase (SEAP) under the control of SV40 promoter (pSEAP2, secreted alkaline phosphatase, Clontech) by use of 40-kDa linear polyethylenimine reagent (PEI-40, Sigma, Munich, Germany). On the third day, the cell culture medium was exchanged for medium without FBS, the IgG preparations of BMP7-aAB positive or negative patients were added, and the cells were stimulated with rhBMP7 or rhBMP2 (0.5 nM, f.c.). The experiments were performed in sextuplicates for BMP7 stimulation and in triplicates for BMP2 stimulation. After 24 h of stimulation, the supernatants were collected and SEAP activity was determined using the SEAP substrate Tropix CSPD (Applied Biosystems) pre-diluted 1:5 in substrate buffer (100 mM Tris, pH 9.5, 100 mM NaCl, 5 mM Mg^2 +^). After 20 min of incubation, SEAP activity (relative light units, RLU) was measured for 2 s per well in a plate luminometer (Mithras LB940, Berthold). BRE reporter activity was determined after cell lysis from the homogenates by injection of 30 μl firefly luciferase substrate (25 mM glycyl-glycine, 7.5 mM MgSO_4_, 2 mM EGTA, 7.5 mM KPO_4_, pH 7.8, 5 mM DTT, 1 mM ATP, 0.1 mM luciferin). Relative light units (RLU) were measured for 2 s, normalized to the according SEAP activity and compared to the unstimulated control.

### 2.5 Statistical analysis

The data were analyzed by SPSS 19.0 (SPSS Inc., Chicago, IL, USA) or GraphPad Prism version 5.01 for Windows (GraphPad Co., La Jolla, CA, USA). Samples were classified as positively containing BMP-aAB (autoantibody positivity) when the measured signals exceeded a floating cut point calculated by adding 1.5 times the inter quartile range (IQR) of the sample cohort to the value defining the 75th percentile (P75 + 1.5 × IQR). This definition is relatively robust and applicable to data sets containing many or no positive samples, as outliers above a certain background noise are reliably and specifically detected. Kolmogorov-Smirnov test for normality of signal distribution was performed and normal distribution was verified for all conditions tested. A *t*-test was performed for calculating the effect of BMP-aAB on the signaling efficacy of rhBMP7 in vitro.

## 3 Results

### 3.1 Assay establishment and characterization

**Fig. 1:**
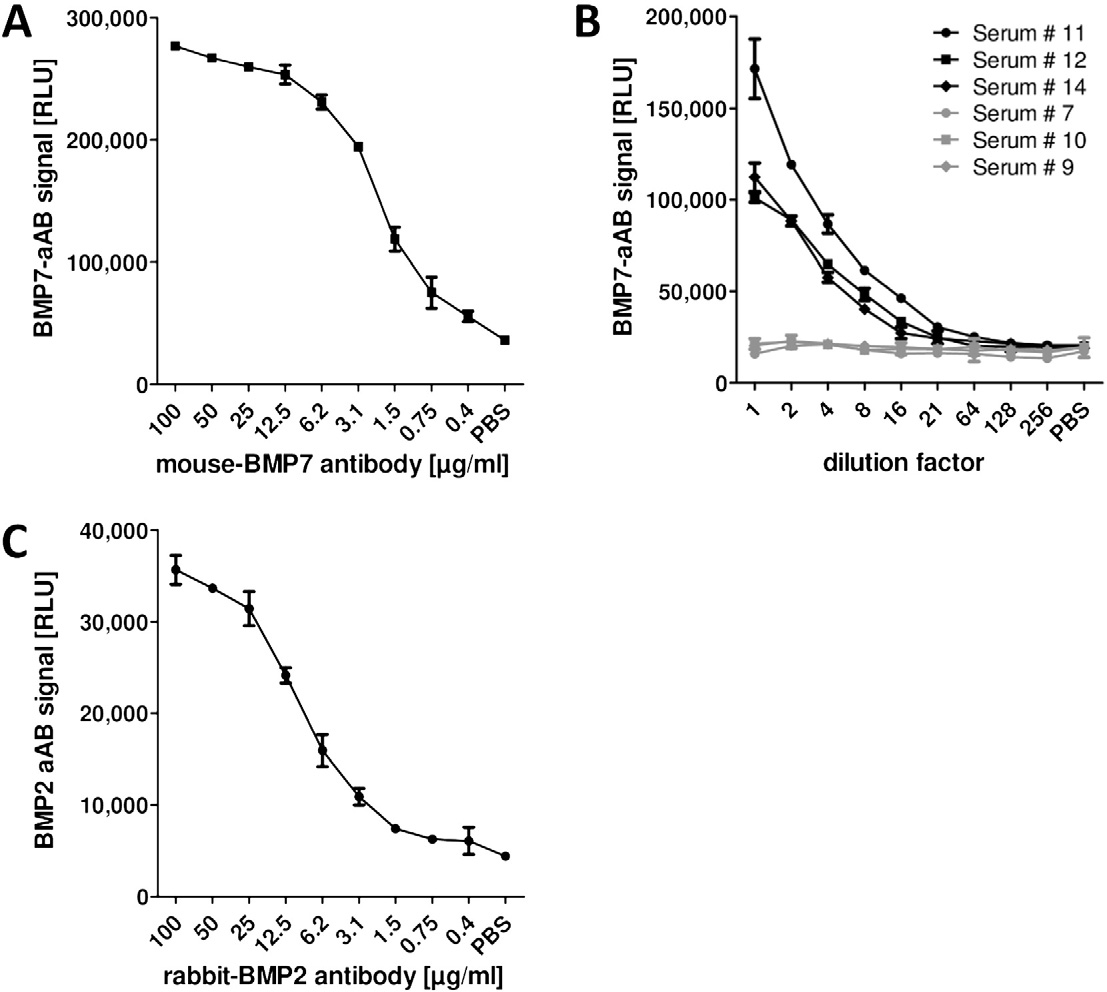
Assay characterization. Linearity and reproducibility of the luminometric signal was tested by (A), using a monoclonal BMP7 antibody; (B), by serial dilutions of positive and negative serum samples (positive, black; negative, grey); and (C), by a commercial polyclonal antibody against BMP2. Nearly linear signals spanning a wide range of concentrations were recorded for both the monoclonal antibody and the BMP7-aAB positive sera derived from the cohort of fracture patients (# 11, 12, 14). The signals obtained from the negative sera (# 7, 10, 9) identified in the same cohort of fracture patients are constant over the whole dilution range. (C) The BMP2-aAB assay was tested by a serial dilution of a polyclonal BMP2 antibody yielding a similar detection range as in the BMP7 assay. n = 2; mean ± SD.

The novel autoimmune assays rely on labelled rhBMP7 or rhBMP2 as bait for the aAB, and on the isolation of the aAB-rhBMP7 or aAB-rhBMP2 complexes from human serum by protein A-mediated precipitation of IgG. After washing the protein A pellet containing the labelled BMP bound by IgG, the chemiluminescence measured directly correlates to the amount of BMP7-aAB or BMP2-aAB present in the sample. In order to generate a positive control, mice were immunized with rhBMP7, hybridoma cells were generated by a commercial partner, positive cells were identified, expanded, and monoclonal antibodies against rhBMP7 were isolated. One monoclonal antibody was chosen for reasons of specificity and used to characterize the BMP7-aAB assay. Signal intensity correlated to monoclonal antibody concentrations over a dilution range from 0.4–25 μg/ml, i.e., over almost two orders of magnitude (Fig. 1-A). Similarly, when comparing BMP7-aAB positive and negative sera, signal intensities correlated to aAB concentrations in diluted samples over more than one order of magnitude (Fig.1-B). Signal intensities of the three aAB-negative samples were highly reproducible with little signal variations over the dilution range and yielded comparable RLU to the PBS background control (Fig.1-B). The BMP2-aAB assay was verified with a commercial anti-BMP2 antibody (Fig. 1-C). These results indicate a successful establishment of novel assays for detecting and quantifying human BMP2 or BMP7 autoimmunity.

### 3.2 Prevalence of BMP7- and BMP2-aAB in control subjects

**Fig. 2:**
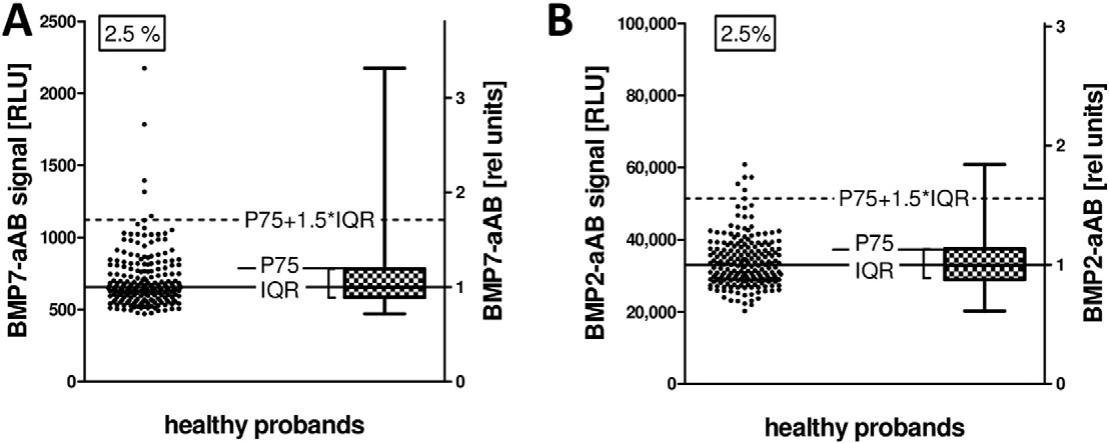
BMP7- and BMP2-aAB measurement in healthy probands. A set of 200 control subjects (n = 100 healthy men and n = 100 healthy women, age range 21–40 years) were analyzed for BMP7- and BMP2-aAB. Samples are classified as aAB positive when the luminometric signal exceeds the P75 + 1.5 IQR cut point (dashed lines). The median is indicated by the solid line, and is set as 1 relative unit of BMP7- or BMP2-aAB. (A) Five samples (2.5%) exceed the cut point and are considered BMP7-aAB positive. (B) Five samples (2.5%) exceed the cut point and are considered BMP2-aAB positive.

The presence of naturally occurring BMP7- and BMP2-aAB was tested in a cohort of 200 healthy men and women. Using the 75th percentile (P75) plus 1.5-times the interquartile range (IQR) as criterion for positivity, five out of 200 samples (2.5%) were classified as containing BMP7-aAB (Fig. 2-A), and a similar prevalence for BMP2-aAB was determined (Fig. 2-B). Four of the BMP7-aAB positive probands were female and one was male, with an age of 25, 37, 38 (2 ×), and 39 years, respectively. The BMP7- and BMP2-aAB signals were of moderate strength, and the RLU values exceeded the median signal intensity by 2–5 times on average. On the basis of these data, relative units of BMP7- and BMP2-aAB titers were deduced in relation to the median (Fig. 2).

### 3.3 Prevalence of BMP7- and BMP2-aAB in patients with bone fractures

**Fig. 3:**
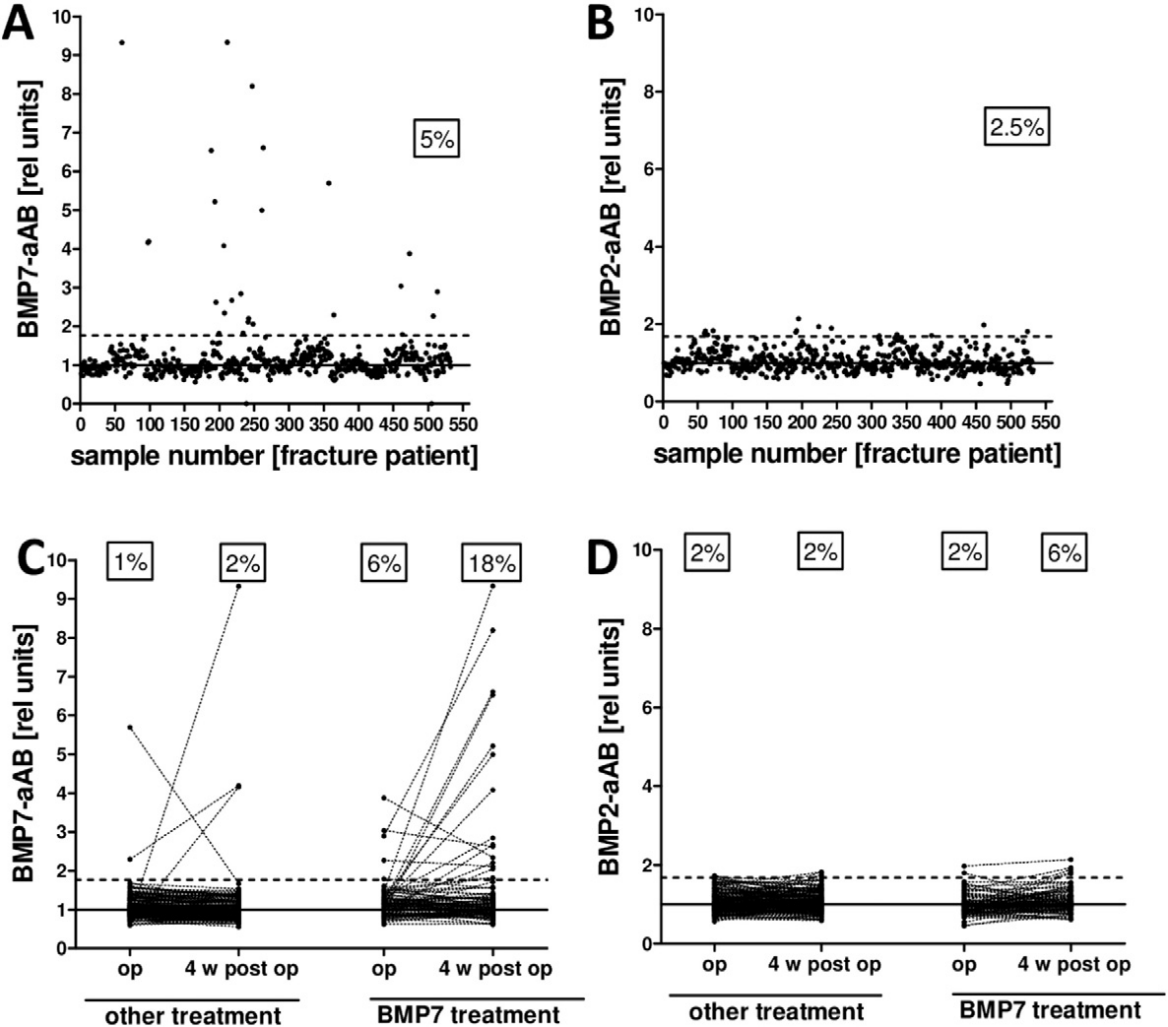
BMP7- and BMP2-aAB measurements in fracture patients. Analysis of 530 samples from 265 fracture patients treated or not with rhBMP7. (A) Of all the measured samples, 5% show signals above the cut point (dashed line) indicating positivity for BMP7-aAB. Some of the BMP7-aAB signals are up to 10 times above the median. (B) The respective number of samples positive for BMP2-aAB is considerably smaller. (C) The prevalence of BMP7-aAB is higher in the BMP7 treated group with 6% positive patients at surgery and 18% four weeks after surgery compared to the untreated group with 1–2% BMP7-aAB positive sera at these time points. Dotted lines connect the samples for a given patient from time point surgery to 4 weeks after surgery. (D) BMP2-aAB detection divided by treatment groups showed 2% of aAB positive samples in the BMP7-naïve group both before and after surgery. Four weeks after BMP7 treatment, the number increased to 6% BMP2-aAB positive patients, albeit without strongly positive reactions.

The same assay and criterion for positivity were used in an analysis of BMP7- or BMP2-aAB in fracture patients. In the collection of 530 serum samples (265 patients measured at two time points), a total of 28 (5%) samples yielded RLU above the cut point of the BMP7-aAB assay (Fig. 3-A), and 14 samples (2.5%) were positive in the BMP2-aAB assay (Fig. 3-B). In comparison to the BMP7-aAB signals detected from the healthy subjects, the positive signals were higher for the fracture patients exceeding the median RLU signal by up to 10 times. This was not the case for the BMP2-aAB signals. According to the therapeutic interventions, patients were divided into rhBMP7-treated (n = 87 patients) or rhBMP7 naïve subjects (n = 178 patients) (Fig. 3-C). In the rhBMP7 naïve group, 2 patients (1% of the subgroup) were found BMP7-aAB positive at surgery and 3 patients (2%) four weeks after the surgery. In the rhBMP7 treated group, a higher prevalence was found, i.e., 5 patients (6%) were measured as BMP7-aAB positive at surgery and 16 patients (18%) four weeks later. In this latter group the average signal intensity of the positive samples was also considerably higher compared to the rhBMP7 naïve group. The majority of patients (n = 244) were tested negative throughout the study, while 5% of the patients (n = 14) newly developed BMP7-aAB (Fig. 3-C), whereas no samples were highly positive for BMP2-aAB, independent of BMP7 treatment (Fig. 3-D). The number of samples with cross-reaction towards both BMP7 and BMP2 was relatively small (n = 4).

### 3.4 Variations of BMP7-aAB concentrations with time

**Fig. 4:**
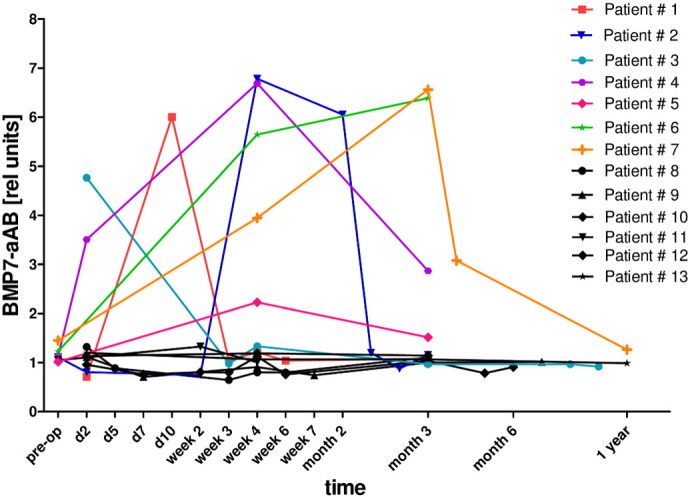
BMP7-aAB trend over time. The patients that were tested positive for BMP7-aAB showed a transient occurrence of aAB. In most cases BMP7-aAB positivity returned to undetectable levels within two months. In one patient (Patient # 6) a decline was not observed in the samples available for analysis (green). The negative control sera from the same study showed a steady signal at background level (black). BMP7-aAB positive sera (Patients 1–7); BMP7-aAB negative sera (Patients 8–13). (For interpretation of the references to color in this figure legend, the reader is referred to the web version of this article.)

To study the persistence of BMP7-aAB titers over time, seven positive and six negative subjects were selected based on signal strengths and availability of longitudinal serum samples, and between three and nine additional time points were analyzed (Fig. 4). All of the patients originally classified as negative based on their pre- and post-surgical sample remained negative at all the additional time points analyzed. The patients that were tested positive for BMP7-aAB in the initial screen either at the time point before surgery or after surgery or at both time points showed a varying pattern of BMP7-aAB titers. In general, most of these patients developed a peak of aAB titers in the first days and weeks after surgery that proved dynamic and transient, returning to baseline levels within the time range analyzed. One patient showed constantly increasing BMP7-aAB titers with time (Patient 6, green), however, only three time points up to 3 months after surgery were available from this patient, and therefore the potentially transient nature of the aAB peak could not be verified in this subject.

### 3.5 Clinical treatment success in relation to BMP7 autoantibodies

**Fig. 5:**
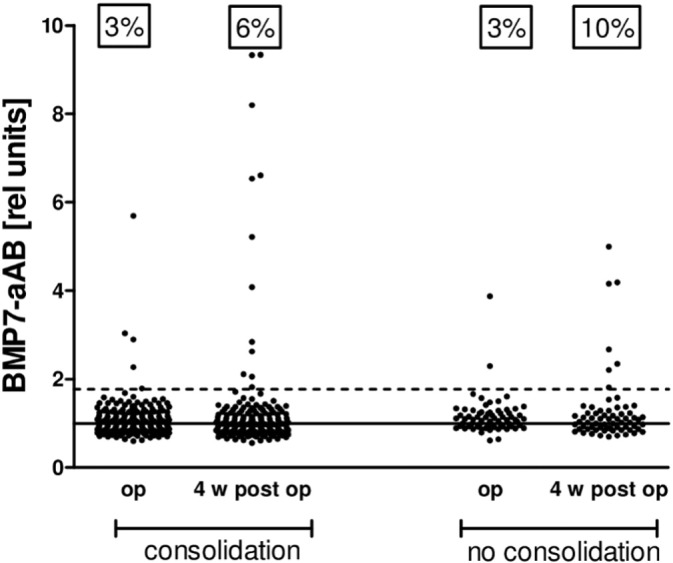
BMP7-aAB concentrations and fracture healing outcome. Fracture patients were divided into those that had a positive healing outcome (consolidation) and those in whom the treatment failed (no consolidation). At surgery both groups showed a prevalence of BMP7-aAB positive samples of 3%. The prevalence of BMP7-aAB positive subjects was higher 4 weeks after surgery, especially in the group where no consolidation was diagnosed. However, highest BMP7-aAB titers were detected in samples from successfully treated patients who developed fracture gap closure and complete consolidation.

The group of fracture patients analyzed is very heterogeneous in terms of fracture type, previous therapeutic interventions, general health status, age, sex, site of injury and other clinical parameters. Nevertheless, in order to get a first impression on the therapeutic importance of the BMP7-aAB, the parameter consolidation describing whether the fracture healing process was successful or not in closing the fracture gap by newly formed bone determined by X-ray, was chosen for analysis. The relative number of BMP7-aAB positive patients is higher in the group of patients without consolidation as compared to successfully treated patients (Fig. 5). However, the samples with the highest relative BMP7-aAB titers are found in the successfully treated group. These findings indicate that BMP7-aAB do not preclude successful consolidation in general.

### 3.6 Biological activity of BMP7-aAB in vitro

**Fig. 6:**
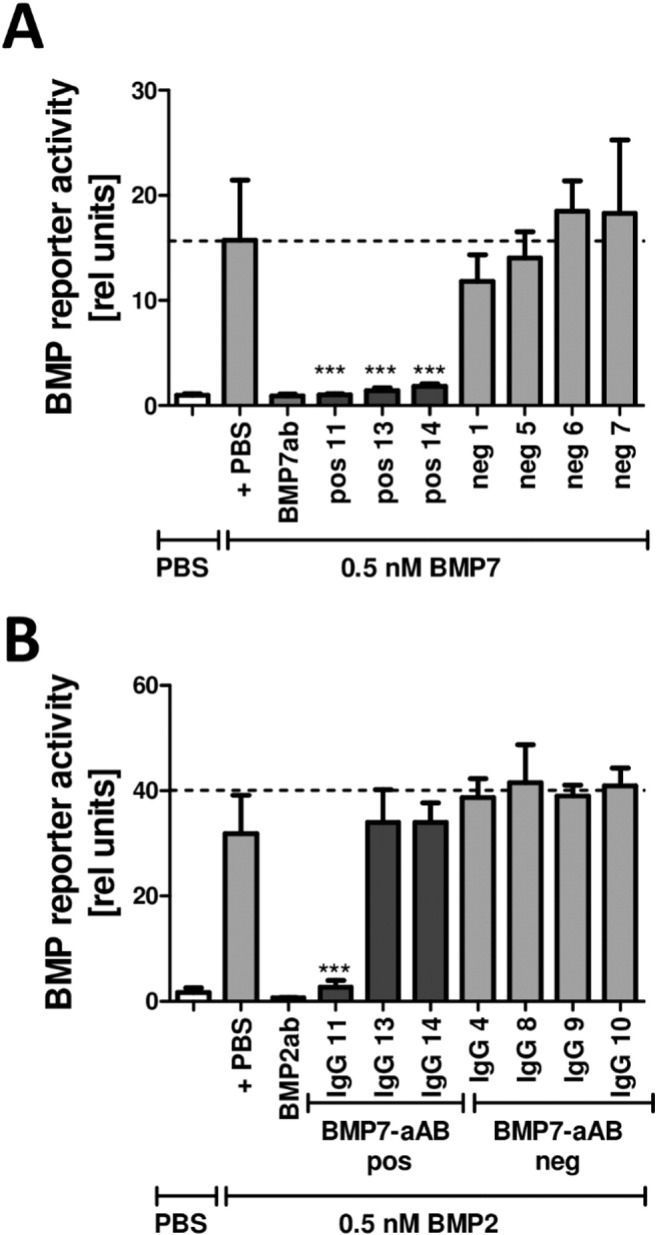
Biological activity of BMP7-aAB. IgG preparations from BMP7-aAB positive and negative serum samples were analyzed using the BRE-luciferase reporter system. NIH3T3 cells were transfected with BRE reporter construct and pSEAP2 for normalization. (A) Transfected cells react to stimulation with 0.5 nM rhBMP7 by firefly luciferase expression. The wells were co-incubated with either a monoclonal BMP7-antibody (BMP7ab) as positive control, or IgG preparations from BMP7-aAB positive (pos 11, 13, 14) or negative (neg 1, 5, 6, 7) serum samples. Co-incubation with BMP7-aAB positive IgG preparations blocked the signal in all three cases, as does the monoclonal BMP7ab. Co-incubation with IgG preparations from BMP7-aAB negative subjects had no effect on BMP7 signaling. (B) Using the same cell culture BMP reporter system, co-incubation with BMP2 induced a luciferase signal which was inhibited by co-incubation with a polyclonal BMP2-antibody (BMP2ab). Co-incubation of BMP2 with IgG from BMP7-aAB positive samples suppressed the BMP2 signal in one (IgG 11) of three cases. No effect on the reporter activity was detected for the other two BMP7-aAB positive or the BMP7-aAB negative preparations. n = 6 (BMP7); n = 3 (BMP2); mean + SD; Student's *t*-test; ***, P < 0.0001.

In order to test whether the human BMP7-aAB are biologically active, an in vitro reporter assay was established with a BRE reporter plasmid and conducted with IgG preparations from the human serum samples. NIH3T3 cells were transfected with the BRE reporter and pSEAP2 for normalization, and rhBMP7 or rhBMP2 was applied as reporter stimulus (Fig. 6-A, -B). PBS served as solvent control and did not activate the reporter, while rhBMP7 (0.5 nM f.c.) or rhBMP2 (0.5 nM f.c.) induced reporter gene activity > 15-fold over control. As positive control for BMP7-specific immunoglobulins, the monoclonal BMP7 antibody (BMP7ab) was applied (Fig. 6-A). Co-incubation of rhBMP7 with BMP7ab completely blocked the reporter signal highlighting its antagonistic activity. Next, IgG preparations from three different BMP7-aAB positive and four BMP7-aAB negative sera from the cohort of fracture patients were applied. Reporter signal strength was completely repressed to basal levels by all three BMP7-aAB positive samples, similar as seen for the BMP7ab, whereas the IgG preparations from the BMP7-aAB negative subjects were without effect. In a second set of experiments, the interaction of BMP7-aAB with BMP2-dependent signaling was tested. As positive control for BMP2-specific immunoglobulins, a polyclonal BMP2 antibody (BMP2ab) was applied (Fig. 6-B). Again, the co-incubation of rhBMP2 with BMP2ab completely blocked the reporter signal. When the same IgG preparations from the three BMP7-aAB positive sera mentioned above were tested, no consistent inhibition of BMP2-stimulated reporter gene activity was recorded. Only one of the positive samples (IgG 11) suppressed the BMP2-stimulated activity, while all the other positive or negative IgG preparations were without effect. Collectively, these results indicate that there is no general cross-reactivity of BMP7-specific aAB to the BMP2 pathway, but that cross-reactivity in certain subjects may occur. In this case, the patient showing positive immunoreactivity against both BMP2 and BMP7 (number 11) was successfully treated and experienced full consolidation of the fracture.

## 4 Discussion

In this study, two novel assays for the detection and quantification of aAB against BMP7 and BMP2 are described. The assays are reproducible, the signals are linear upon dilution of control antibodies, and the assays have been validated by specific antibodies against BMP7 and BMP2, respectively. Using a robust and prudent criterion for positivity, we have detected BMP7- and BMP2-aAB both in healthy controls and in fracture patients. The general recommendations on how to develop aAB assays and test the functionality of aABs have been taken into account during the course of this study [22], [23], [24], [25].

The difference in prevalence of BMP7-aAB observed between patients treated or not with rhBMP7 further supports the notion that this novel assay is capable of measuring both naturally occurring and rhBMP7-induced aAB against BMP7. Final proof of the reliability of the novel assay is given by the functional characterization of IgG preparations from human serum samples, indicating that only BMP7-aAB positive preparations were capable of antagonizing BMP7-signaling, but not BMP7-aAB negative preparations. Moreover, most of the IgG preparations from BMP7-aAB positive samples (two of three) were not capable of suppressing BMP2-stimulated reporter gene activity, verifying the specificity of these aAB. These results highlight the quality of the novel BMP7-aAB assay, and indicate that the BMP7-aAB detected by the novel assay are capable of reacting with recombinant human BMP7 and interfering with BMP7 signaling.

The prevalence of 2.5% for BMP7-aAB in healthy adults is in line with a previous study that described a prevalence of 1.6% and 3% of naturally occurring aAB against BMP7 in subjects younger or older than 65 years of age [26]. Collectively, these data indicate that a small fraction of healthy adults are already positive for BMP7-aAB despite not having been exposed to rhBMP7 before in their life. In contrast to our analysis, the former study reported a prevalence of up to 50% in BMP7-treated patients [26]. This value is much higher than observed in our study, where up to 18% of patients proved positive for BMP7-aAB after rhBMP7 exposure. The reasons for this discrepancy are unclear, but might lie in the different treatment regimen applied, the different assays used or the criteria applied for defining positivity.

Both studies highlight that natural BMP7-aAB exist and that BMP7-aAB can be induced by rhBMP7 treatment. This autoimmune response to the applied rhBMP7 does not develop in all of the treated patients, but in a subset. The parameters predisposing some patients to BMP7-aAB are probably similar to those determining the risk of autoimmunity in the general population, including genetic predisposition [27], obesity [28], smoking status [29], and other environmental factors. Specifically, B-cells are primed to be activated upon a sudden increase in antigen along with inflammatory signals (cytokines) whereas constant antigen signals in absence of inflammation may rather be tolerated [30], [31]. The application of rhBMP7 in fracture patients provides an unprecedented antigen surge into an inflamed environment. Patients that contain autoreactive B-cells which are in immunological tolerant form may become activated especially in the context of an ongoing inflammation. These aspects as well as the genetic predisposition to develop autoimmunity vary between individuals and may account for the heterogeneous picture observed. Further studies along this line are needed.

Comparing the aAB against BMP2, the former study reported a prevalence of 2.3% in subjects below 65 years of age, and 5.3% in subjects who are older [26]. Again, these data are well in agreement with our results in patients and controls. Notably, the prevalence of BMP2-aAB positive patients was higher in samples collected after rhBMP7 treatment, however the titers of these aAB were relatively low and close to the cut-off level. These data imply a high specificity of therapy-induced aAB to the actual biological agent used with little cross-reactivity to a related growth factor.

When reconsidering the three main concerns regarding anti-drug antibodies of biologicals, i.e., (1) neutralization of the drug, (2) immune reaction by the drug-aAB complexes, and (3) cross-reactivity of the aAB to the endogenous protein, the first issue seems to apply to therapy-induced BMP7-aAB as they neutralized the activity of the recombinant protein in vitro and prevented it from activating its signaling cascade. However, whether the neutralizing effect has any strong and relevant clinical consequences could not fully be evaluated in this study, due to the heterogeneity and limited number of aAB-positive subjects. Analyzing consolidation as the most relevant end-point of the treatments, our data indicate that indeed there was a higher number of BMP7-aAB positive patients who failed to reach successful fracture gap closure, however the subjects with the highest BMP7-aAB titers were found in the group of successfully treated patients. These results are puzzling and do not allow a clear-cut statement on the clinical importance of the natural or therapy-induced BMP7-aAB, yet. Cross-reactivity of the BMP7-aAB to the endogenous protein is likely to occur because the drug is a recombinant human protein variant with highest similarity to the endogenous form. However, the short transient nature of the induced BMP7-aAB peaks argues against adverse long term consequences, except for situations in which the antigen is again provided, e.g. upon repeated therapy with the biological in case that fracture healing failed.

Antibody formation against biologicals has long been known, e.g. during the course of interferon-β (IFN-β) [32], [33], [34], erythropoietin (EPO) [35] or thrombopoietin (THPO) treatment [36]. In all of these applications, it was shown that aAB are capable of interfering with the efficacy of the drug. EPO antibodies were associated with the severe consequence of pure red cell aplasia [35]. Antibody development against THPO was associated with thrombocytopenia due to cross-reaction with the endogenous THPO. The incidence of thrombocytopenia was higher in healthy volunteers than in immune compromised cancer patients, and similar to our study, also the antibodies against THPO disappeared over time [36]. Anti-IFN-β antibodies are extensively studied to better characterize their occurrence and effects [26], [37], [38]. However, the mechanisms underlying the patient-specific responses to the biologicals are still far from being understood. All of the treatments mentioned above are given systemically and over a longer period of time, in contrast to the rhBMP7, which is applied locally into the fracture gap usually only once. Here, a relatively high amount of rhBMP7 is suddenly present initiating a fast biological response but its stability and the actual amount that is actively involved in bone regeneration is difficult to estimate. Similarly, the aAB induced by the treatment are not characterized by a stable titer and are equally difficult to be assessed for their potentially interfering capacity and clinical importance.

## 5 Conclusions

Treatment with rhBMP7 triggers bone formation along with a transient autoimmune response in some patients. The induced BMP7-aAB are neutralizing in nature, as seen in the in vitro analysis, and may therefore potentially affect the osteogenic differentiation. This phenomenon may be of clinical importance as some patients fail to respond to the osteoinductive treatment for as yet unknown reasons. However, the correlation of treatment success (consolidation) with the BMP7-aAB positivity and BMP7-aAB concentrations failed to provide a clear-cut picture for a negative role in bone regeneration in our study. Collectively, the transient nature of these therapy-induced BMP7-aAB, the lack of a general cross-reactivity to BMP2, and the lack of a clear correlation to treatment failure are re-assuring findings with respect to the safety of recombinant BMP in fracture healing. It remains to be analyzed in future studies whether therapy-induced BMP7-aAB are a problematic predisposition for a repeated BMP7 treatment in cases of initial treatment failure. We hope that our herein described analytical assays, which are straightforward, robust and easy to establish, are of help in further studies addressing this issue.

## Conflicts of interest

All authors declare that no conflict of interests exists.

## Source of funding

This study was financially supported by the Charité-Universitätsmedizin, DFG (FOR 2165), and the Berlin-Brandenburg Center for Regenerative Therapies (BCRT) (13GW0099). Andrea Schuette is a member of the DFG funded Berlin-Brandenburg School for Regenerative Therapies (BSRT) GSC 203. The sponsors had no role in study design; in the collection, analysis and interpretation of data; in the writing of the report; and in the decision to submit the article for publication.

## Transparency Document

Transparency document. Image 2

## Figures and Tables

**Table 1:** Study design. Fracture patients were divided into two groups (treated or not with BMP7) that received differential surgical treatments.

BMP7 treatment	Patients [n]	Treatment groups	Patients [n]	BMP7-aAB over time, patients [n]
Fracture treatment w/o BMP7	178	Fresh fractures	145	5
		Pseudarthrosis	33	1
Fracture treatment with BMP7	87	Pseudarthrosis	38	2
		Pseudarthrosis RIA^a^ or Spongiosa	21	2
		Pseudarthrosis Masquelet^b^	28	3

a RIA, reamer irrigator aspirator.

b Masquelet, use of temporary cement spacer.
